# Quantification of the Hardened Cement Paste Content in Fine Recycled Concrete Aggregates by Means of Salicylic Acid Dissolution

**DOI:** 10.3390/ma15093384

**Published:** 2022-05-09

**Authors:** Zengfeng Zhao, Jianzhuang Xiao, Denis Damidot, Sébastien Rémond, David Bulteel, Luc Courard

**Affiliations:** 1 Department of Structural Engineering, College of Civil Engineering, Tongji University, Shanghai 200092, China; zengfengzhao@tongji.edu.cn; 2 Univ. Lille, Institute Mines-Télécom, Univ. Artois, Junia, ULR 4515—LGCgE—Laboratoire de Génie Civil et géo-Environnement, F-59000 Lille, France; denis.damidot@imt-nord-europe.fr (D.D.); david.bulteel@imt-nord-europe.fr (D.B.); 3 IMT Nord Europe, Institut Mines-Télécom, Centre for Materials and Processes, F-59000 Lille, France; 4 University Orléans, University Tours, INSA CVL, LaMé, EA 7494, F-45100 Orléans, France; sebastien.remond@univ-orleans.fr; 5 Building Materials, Urban and Environmental Engineering, University of Liège, 4000 Liege, Belgium; luc.courard@uliege.be

**Keywords:** hardened cement paste content (*HCPC*), recycled concrete aggregates (RCA), salicylic acid, water absorption, X-ray Diffraction, density

## Abstract

Adherent hardened cement paste attached to recycled concrete aggregates (RCA) generally presents a higher porosity than natural aggregates, which induces a lower porosity in the properties of RCA. The characterization of the adherent hardened cement paste content (*HCPC*) in the fine RCA would promote better applications of RCA in concrete, but the determination of *HCPC* in fine RCA is not well established. A simple method based on salicylic acid dissolution was specifically developed to quantify the *HCPC* in RCA, especially for RCA containing limestone aggregates. The results demonstrated that the soluble fraction in salicylic acid (*SFSA*) was equal to the *HCPC* for white cement and slightly lower for grey Portland cement, which was also confirmed by a theoretical approach using modelling the hydration of cement paste with the chemical equations and the stoichiometric ratios. The physical and mechanical properties of RCA (e.g., water absorption) were strongly correlated to the *SFSA*. For industrial RCA, *SFSA* did not give the exact value of *HCPC*, but it was sufficient to correlate *HCPC* with the other properties of RCA. The water absorption could be estimated with good accuracy for very fine RCA (laboratory-manufactured RCA or industrial RCA) by extrapolating the relationship between water absorption and *HCPC*, which is very important for concrete formulation.

## 1. Introduction

A huge amount of construction and demolition waste (CDW) is generated annually (e.g., the European Union generated 838.9 million tons of CDW in 2021 according to Eurostat [1]). On the other hand, good quality aggregate (e.g., the European Union of 39 countries: 3.07 billion tons of aggregates in 2018 [2]) is needed for the construction industry according to European Aggregates Association. Recycling CDW as aggregate in the concrete industry would be a partial solution to the aggregate shortage and waste disposal problem [3,4]. The main components of CDW are old concrete (ranging from 32% to 75%), bricks, wood from buildings, glass, gypsum asphalt from the pavement, plastics, etc. [5,6]. The use of RCA crushed from CDW to replace the natural aggregates (NA) in the production of concrete has increased over the last decade [7,8,9,10,11].

RCA is generally composed of a mix between NA and adherent mortar (or adherent hardened cement paste) and the complete separation of them is quite difficult [12]. Hardened cement paste presents a much higher porosity than the NA generally used for the manufacture of concrete [13,14]. Therefore, the quantity and the quality of hardened cement paste are responsible for the poorer properties of RCA, such as higher porosity [15], higher water absorption [13,15], lower density [15,16], and lower resistance to crushing and abrasion [13]. Concrete produced with RCA generally shows lower workability [9,17], lower mechanical behavior [11,18,19], and lower durability performance than NA-based concrete [17,18,19,20,21,22]. The effect of *HCPC* is even more crucial for fine RCA (FRCA: fraction of RCA less than 5 mm), which makes them difficult to apply into mortar or concrete compared with coarse RCA (CRCA: fraction of RCA greater than 5 mm) [10,14,23,24].

To promote better application of RCA in the concrete, it is important to study the role of *HCPC* in RCA despite the quantitative influence of *HCPC* on the physical and mechanical properties of RCA is not well described.

Currently, there is no standard to determine *HCPC* or adherent mortar content in RCA; however, some methods were developed in the literature (Table 1). The thermal method [25] and the sodium sulphate solution method are only applicable to coarse RCA because the removal of mortar is difficult with small particles, and they are only suitable for determining the adherent mortar content of coarse RCA. The image analysis method [26] and the linear traverse method [27,28] are suitable for the quantification of residual mortar in coarse RCA. The X-ray SEM-based image analysis developed by Ulsen et al. [29] is too long to perform as a statistical approach is needed to obtain reliable results. The deionized water dissolution has the carbonation contamination problem. It also takes ten days, which is too long, and the ICP analysis is expensive [30]. For the hydrochloric acid solution method, it cannot be applied for RCA made with limestone, which is soluble in hydrochloric acid [31,32]. The above-mentioned methods seem to be not adapted to the characterization of *HCPC* in fine RCA, especially for RCA containing limestone [33,34,35].

*HCPC* in fine RCA is closely related to other physical and mechanical properties (e.g., density and water absorption) of fine RCA, which play an important role in concrete formulation [23,36]. Indeed, the water absorption of fine RCA has to be accurately quantified to assess the effective water used in concrete [37,38,39,40,41,42]. However, the current methods (such as EN 1097–6 [43] or ASTM C 128 [44], IFSTTAR No. 78 method [45]) used to determine the water absorption of fine RCA are generally not accurate, particularly for the RCA containing a high percentage of fine particles [46,47].

This paper intends to develop and validate an easily performed method to quantify *HCPC* in fine RCA by a theoretical approach and an experimental method. In addition, the validity of the method was conducted by applying it to industrial produced RCA, laboratory-manufactured noncarbonated RCA, and well-carbonated RCA. The relationship between the main properties of fine RCA and HPCP was correlated and a new method to determine the water absorption of industrial produced and laboratory-manufactured fine RCA was proposed.

## 2. Materials and Methods

### 2.1. Laboratory-Produced RCA

Laboratory-produced initial concrete (named OC1, OC2 and OC3) with two different water-to-cement (W/C) ratios (W/C = 0.6 for OC1 and OC2, W/C = 0.4 for OC3) and the volumes of paste (278 dm^3^/m^3^ for OC1, around 350 dm^3^/m^3^ for OC2 and OC3) were manufactured. The fine RCA was obtained by crushing the concrete after 28 and 90 days of curing using a jaw crusher. These three concretes were prepared with white cement (CEM I 52.5 N according to EN 197–1 [48] from Lafarge company: Teil factory, France) and limestone aggregates (sourced from Tournai, Holcim France Benelux). A total of 1138 kg of aggregate, 756 kg of sand and 299 kg of cement were used for the concrete OC1 while 1041 kg of aggregate, 692 kg of sand and 376 kg of cement were used for the concrete OC2. For the concrete OC3, 1019 kg of aggregate, 677 kg of sand and 475 kg of cement were used. More detailed information on prepared concrete can be found in Zhao et al. [49]. The fraction 0/5 mm of RCA was obtained after crushing and they were divided into four granular classes: 0/0.63, 0.63/1.25, 1.25/2.5, and 2.5/5 mm. They were pre-dried at 105 °C and then stored in sealed bags to minimize carbonation. These laboratory-manufactured noncarbonated RCA were noted as RCAl_OCx_28/90 (x refers to the type of three initial concretes). To study the influence of carbonation, 1 kg of these different granular classes of RCAl_OC1_90 was then stored in the accelerated carbonation chamber for two weeks (pure CO_2_ at 20 °C and relative humidity of 75% were used to achieve nearly complete carbonation and maximize the effect of carbonation). The carbonation degree of RCA was verified by the phenolphthalein indicator, the colour of RCA after carbonation treatment measured by phenolphthalein was not pink (while it was pink for the RCA stored in natural carbonation condition), which indicated that the sample was well-carbonated after two weeks’ storage in accelerated chamber [12]. All the laboratory-manufactured noncarbonated RCA and laboratory-manufactured well-carbonated RCA (noted as RCAl_OC1_90 wc) were characterized including *HCPC*, density and water absorption. Each granular class of RCA is referred to as its average particle size.

Pure cement pastes with a W/C ratio of 0.5 were prepared and then cured in water for 28 and 90 days. They were made with the previous white cement (CEM I 52.5 N of Lafarge, noted as White cement CEM I 52.5 N) and grey cement (CEM II/A-L 52.5 N according to EN 197-1 provided from Holcim in France, noted as Grey cement CEM II/A-L 52.5 N). Pure cement pastes with a W/C ratio of 0.6 were prepared and cured in water for 500 days with cement CEM III/A 42.5 N and cement CEM I 52.5 N according to EN 197-1 from CBR Company in Belgium (noted as CBR CEM III/A 42.5 N and CBR CEM I 52.5 N, respectively). Table 2 presents the mineralogical compositions of cement using the Rietveld method. To assess the impact of insoluble and soluble phases in salicylic acid and methanol solution, specific experiments were performed on pure cement pastes with a white cement CEM I 52.5 N of Lafarge and a grey cement CEM II/A-L 52.5 N of Holcim. These cement pastes made with a W/C ratio of 0.5 were studied after 28 days of hydration (fraction 1.25/2.5 mm) by X-ray Diffraction (XRD) before and after dissolution, and RCAl_OC1_90 0.63/1.25 mm was also investigated. The samples were measured with XRD using a Bruker D8 Advance diffractometer (with a Co K*α*1 radiation, sweep from 10° to 100° 2θ).

### 2.2. Industrial RCA

Three commercial RCA obtained from industrial recycling plants were investigated together with the laboratory-produced RCA. The first two industrial RCA (noted as RCAi1 and RCAi2) were obtained by the Colas Company in France. The third industrial RCA (noted RCAi3) was provided by the French National Project Recybéton [50]. The same tests on the four granular classes were conducted for the industrial RCA as the laboratory-produced RCA.

### 2.3. Experimental Methods

#### 2.3.1. Water Absorption

The water absorption of fine RCA (each granular class) was measured with two methods: the European standard EN 1097-6 [43] and No. 78 of IFSTTAR [44]. A detailed comparison of these two experimental methods is presented in Reference [49]. The mean value of water absorption was measured from three representative samples.

#### 2.3.2. Density

The specific density of fine RCA was determined using a helium pycnometer (Micromeritics AccuPyc 1330). The representative samples of fine RCA were pre-dried at 105 °C before the density analysis.

#### 2.3.3. Hardened Cement Paste Content

A dissolution of hardened cement paste in salicylic acid and methanol solution method was developed, which can be easily performed in an industrial laboratory. The selective dissolution of hardened cement paste in salicylic acid and methanol solution is indeed well known for estimating the content of blast furnace slag (BFS) in blended cement composed of BFS and ordinary Portland cement (OPC) (dissolving the unhydrated cement and the hydration products, leaving only the blast furnace slag undissolved) [51,52,53].

**Figure 1:**
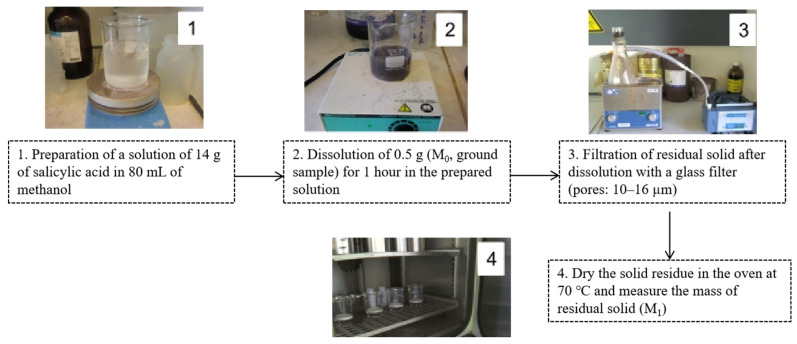
Experimental protocol of measurement of hardened cement paste content in RCA (the subfigure refers to the corresponding procedure).

The principle of this selective dissolution was based on 1 h of dissolution in a solution of 14 g of salicylic acid in 80 mL of methanol [49]. Generally, the representative samples (RCA) were pre-dried in an oven at 105 °C until the constant mass was reached, then they were ground to a particle size of less than 200 µm. Detailed information on the experimental protocol is shown in Figure 1. The soluble fraction in the salicylic acid (*SFSA*) was then calculated as follows (Equation (1)):

(1) $$SFSA\left(\%\right)=\frac{M_{0}-M_{1}}{M_{0}}\times 100$$

where *M*_0_ is the mass of the dried sample before the dissolution and *M*_1_ is the mass of the residual dried sample after the dissolution.

## 3. Results and Discussion

### 3.1. Measurement of Hardened Cement Paste Content in RCA

#### 3.1.1. Experimental Approach

**Figure 2:**
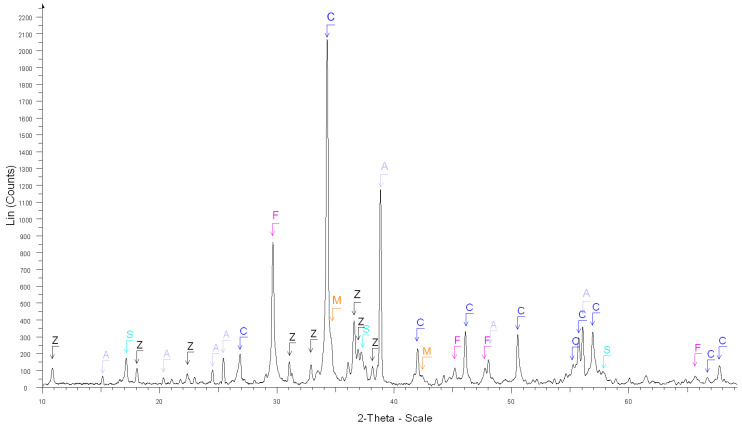
XRD diffractograms: After the dissolution of white cement. C, F, Z, S, A and M stand for calcite, calcium sulfate, syngenite, calcium sulfate hydrate, calcium sulfate hydrate, portlandite and calcite magnesian, respectively.

**Figure 3:**
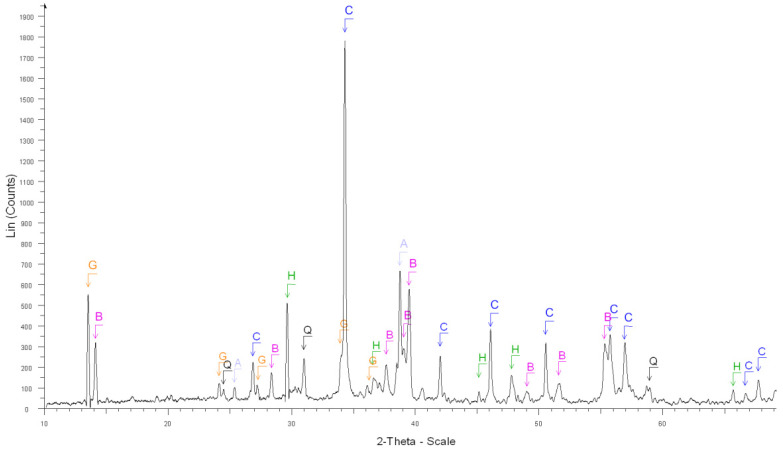
XRD diffractograms: After the dissolution of grey cement. C, B, H, G, Q, A and M stand for calcite, brownmillerite, anhydrite, gypsum, quartz, calcium aluminum oxide and calcite magnesian, respectively.

**Figure 4:**
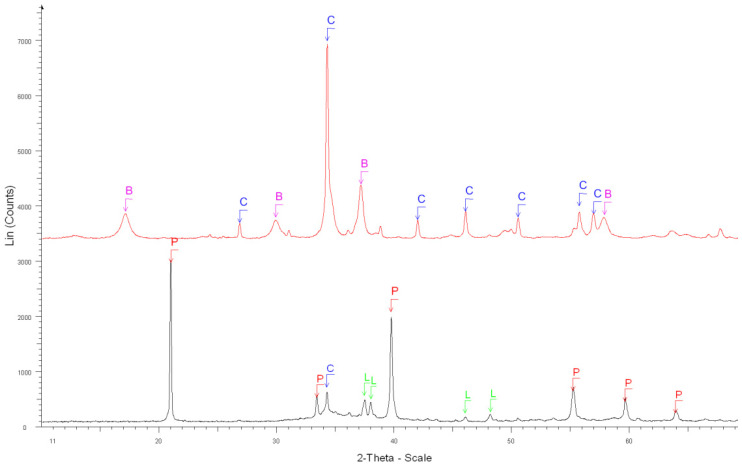
XRD diffractograms: Before (black curve below) and after the dissolution (red curve above) of white cement paste. B, C, P and L stand for brownmillerite, calcite, portlandite and β-dicalcium silicate, respectively.

**Figure 5:**
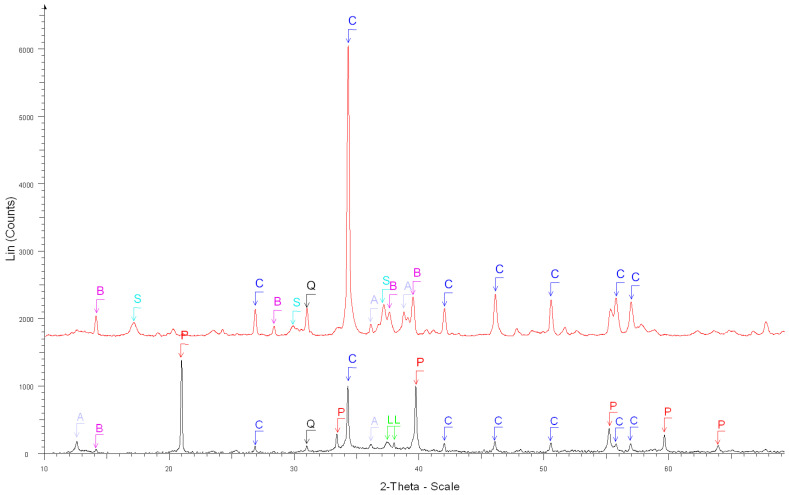
XRD diffractograms: Before (black curve below) and after the dissolution (red curve above) of grey cement paste. B, S, C, Q, A, P and L stand for brownmillerite, calcium sulfate hydrate, calcite, quartz, calcium aluminum oxide, portlandite and β-dicalcium silicate, respectively.

**Figure 6:**
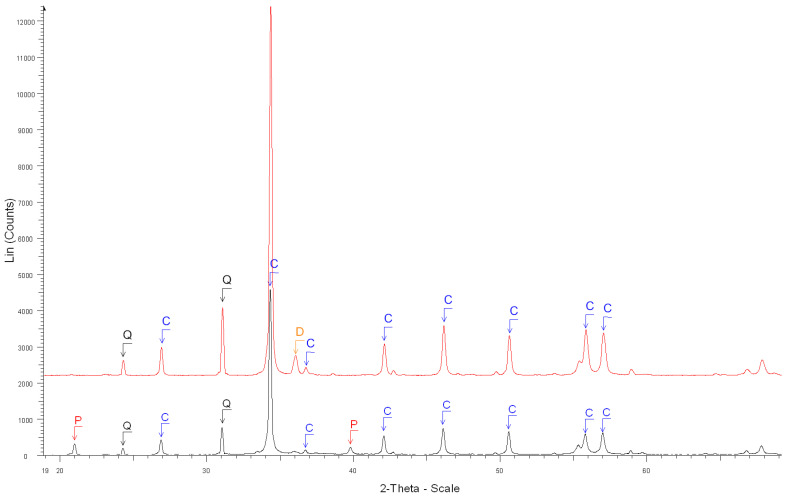
XRD diffractograms: Before (black curve below) and after the dissolution (red curve above) of RCAl_OC1_90 0.63/1.25 mm. Q, C, D, and P stand for quartz, calcite, dolomite and portlandite, respectively.

The accuracy of the estimate of hardened cement paste content (*HCPC*) by the soluble fraction in the salicylic acid (*SFSA*) mostly depends on the amount of soluble versus insoluble phases contained in the cement paste of fine RCA [54]. Table 3 shows the XRD results before and after the dissolution of cement and cement pastes compared with the ICDD database (corresponding XRD diffractograms are shown in Figure 2, Figure 3, Figure 4, Figure 5 and Figure 6).

From these results, most of the phases contained in OPC cement paste (Ca(OH)_2_, C-S-H, ettringite, *C*_2_*S*, *C*_3_*S*, CaO) was soluble in salicylic acid except some phases such as *C*_3_*A*, *C*_4_*AF*, Gypsum, *C*_3_*A*H_6_, AFm and blast furnace slag; however, the quantity of these insoluble phases was relatively low in the hydrated cement paste. The main phases contained in natural aggregates (Quartz, Dolomite, Calcite) were insoluble in salicylic acid. Thus, apart from calcium aluminate phases and their corresponding hydrates, all cement paste is expected to be dissolved.

Two other cement pastes (cement CBR CEM I 52.5 N and CBR CEM III/A 42.5 N, W/C = 0.6, cured in water for 500 days) were also conducted to study the influence of long curing (a cement paste with a high degree of hydration). A crushed calcareous aggregate from France and siliceous sand complying with European standard EN 196-1 [55] were also tested. The experimental results showed that 95.57% of white cement paste and 62.99% of grey cement paste were dissolved while only 0.83% of siliceous sand and 3.21% of calcareous aggregate were dissolved (Table 4). For white cement paste, the *SFSA* was almost identical to the hardened cement paste content (*HCPC*). This was the reason why white cement was chosen for the manufacture of initial concretes. For the grey cement paste (Table 4), the grey cement had a larger content of *C*_3_*A*, *C*_4_*AF* and calcite which do not dissolve in salicylic acid, so *SFSA* corresponded to only 62.99% of *HCPC*. For grey cement paste, *SFSA* was always lower than *HCPC*. For cement pastes made with CBR CEM III/A 42.5 N and CBR CEM 52.5 N after a long time cured in water, *SFSA* corresponded to 80.14% and 78.42%, respectively. Most phases of cement paste made with blended cement (CEM III/A: Portland cement combined with BFS) can dissolve in salicylic acid, which corresponded to 80.14% of *HCPC*. For industrial RCA generally containing a grey cement paste, *SFSA* did not give the exact value of *HCPC*, but it will be demonstrated later that for a given RCA, *SFSA* was sufficient to correlate *HCPC* with the other properties of RCA. This method was also chosen because it was easy to perform, and a very small standard deviation was observed.

#### 3.1.2. Theoretical Approach to the Estimation of *SFSA*

As discussed in Section 3.1.1, *SFSA* was not exactly *HCPC*. However, the difference between *SFSA* and *HCPC* can be determined theoretically through modelling. The chemical equations for modelling the hydration of cement paste (CEM I and CEM II with inert material such as limestone) and their corresponding stoichiometric ratios are listed in Table 5.

The *SFSA* can be estimated based on the degree of hydration *α*, soluble hydrates (C-S-H, CH and ettringite) and the amount of soluble anhydrous cement (*C*_3_*S* and *C*_2_*S*). For the hydration of the aluminate (reaction with gypsum and not with anhydrite), the anhydrite was considered hydrated as gypsum before reacting with aluminates [56]. It was assumed that gypsum and anhydrite were present in the cement in stoichiometric amounts so that the reaction with the aluminate was complete at the maximum degree of hydration. The *SFSA* was determined by Equation (2):

(2) $$SFSA\left(\alpha \right)=\frac{M_{anh,sol}+M_{hydr,sol}}{M_{a}+M_{in}+M_{anh}+M_{hydr}}$$

where *M_anh,sol_* and *M_hydr,sol_* are the soluble masses of anhydrous and soluble hydrates at a degree of hydrations *α*, *M_a_* is the mass of natural aggregates (considered insoluble), *M_in_* is the inert phases in the cement considered insoluble (e.g., calcite), *M_anh_* and *M_hydr_* are the mass of anhydrous cement (*C*_3_*S*, *C*_2_*S*, *C*_3_*A*, *C*_4_*AF*, gypsum and anhydrite) and the mass of hydrates for the degree of hydration *α*, respectively.

The degree of hydration *α* is defined as the fraction of the anhydrous cement that has already hydrated, which relates to the amount of cement consumed (inert phases are not accounted, e.g., calcite). The degree of hydration *α* varies between 0 and 1 whatever the cement composition (Equation (3)).

(3) $$\alpha =\frac{M_{anh,consump}}{M_{anh,0}}$$

Two variables *x* (Equation (4)) and *y* (Equation (5)) were used to quantify the soluble fraction of the cement in the water and the soluble fraction of the cement in the salicylic acid:

(4) $$x=\frac{M_{anh,0}}{M_{ic}}=C_{3}S+C_{2}S+C_{3}A+C_{4}AF+C\bar{S}H_{2}+C\bar{S}$$

(5) $$y=\frac{M_{anh,sol}}{M_{anh}}=\frac{C_{3}S+C_{2}S}{C_{3}S+C_{2}S+C_{3}A+C_{4}AF+C\bar{S}H_{2}+C\bar{S}}$$

where *M_ic_* is the mass of initial cement (*M_ic_* = *M_in,*0*_* + *M_anh,*0*_*), *C*_3_*S*, *C*_2_*S*, … are the mass contents of the various constituents of the cement obtained by using the Rietveld method (Table 2).

It was considered that the composition of anhydrous cement does not change during hydration (the relative proportions of *C*_3_*S*, *C*_2_*S*, *C*_3_*A*, *C*_4_*AF*, gypsum and anhydrite are thus assumed to be constant). Thus, the parameter y is also constant during hydration. Finally, 25% of aluminates formed as ettringite and 75% of aluminates formed as AFm were assumed throughout the hydration. With all the above assumptions, the total mass of anhydrous and the mass of soluble anhydrous in the salicylic acid for a given degree of hydration *α* can be determined, respectively (Equations (6) and (7)):

(6) $$M_{anh}=xM_{ic}\left(1-\alpha \right)$$

(7) $$M_{anh,sol}=yxM_{ic}\left(1-\alpha \right)$$

The mass of soluble hydrates in salicylic acid can be given as Equation (8):

(8) $$M_{hydr,sol}=M_{C-S-H}+M_{CH}+M_{Ett}=\alpha M_{ic}\left\{\begin{array}{l} C_{3}S\left[1+\left(\frac{E}{C_{3}S}\right)\right]+C_{2}S\left[1+\left(\frac{E}{C_{2}S}\right)\right]+\\ 0.25\left\{C_{3}A\left[1+{\left(\frac{E}{C_{3}A}\right)}_{Ett}\right]+C_{4}AF\left[1+{\left(\frac{E}{C_{4}AF}\right)}_{Ett}\right]\right\} \end{array}\right\}$$

We assumed that:

(9) $$C_{sol}=C_{3}S+C_{2}S+0.25\left(C_{3}A+C_{4}AF\right)$$

(10) $${\left(\frac{E}{C}\right)}_{sol}=C_{3}S\left(\frac{E}{C_{3}S}\right)+C_{2}S\left(\frac{E}{C_{2}S}\right)+0.25\left[C_{3}A{\left(\frac{E}{C_{3}A}\right)}_{Ett}+C_{4}AF{\left(\frac{E}{C_{4}AF}\right)}_{Ett}\right]$$

(11) $${\left(\frac{E}{C}\right)}_{moy}=\left\{\begin{array}{l} C_{3}S\left(\frac{E}{C_{3}S}\right)+C_{2}S\left(\frac{E}{C_{2}S}\right)+C\bar{S}\left(\frac{E}{C\bar{S}}\right)+0.25C_{3}A{\left(\frac{E}{C_{3}A}\right)}_{Ett}+\\ 0.75C_{3}A{\left(\frac{E}{C_{3}A}\right)}_{AFm}+0.25C_{4}AF{\left(\frac{E}{C_{4}AF}\right)}_{Ett}+0.75C_{4}AF{\left(\frac{E}{C_{4}AF}\right)}_{AFm} \end{array}\right\}$$

We can obtain:

(12) $$M_{hyd}=\alpha xM_{ic}+\alpha M_{ic}{\left(\frac{E}{C}\right)}_{moy}$$

(13) $$M_{hyd,sol}=\alpha M_{ic}\left(C_{sol}+{\left(\frac{E}{C}\right)}_{sol}\right)$$

Finally, the *SFSA* can be written as Equation (14):

(14) $$SFSA=\frac{yxM_{ic}\left(1-\alpha \right)+\alpha M_{ic}\left(C_{sol}+{\left(\frac{E}{C}\right)}_{sol}\right)}{M_{a}+M_{ic}+\alpha M_{ic}{\left(\frac{E}{C}\right)}_{moy}}$$

#### 3.1.3. Application to Pure Cement Pastes

**Figure 7:**
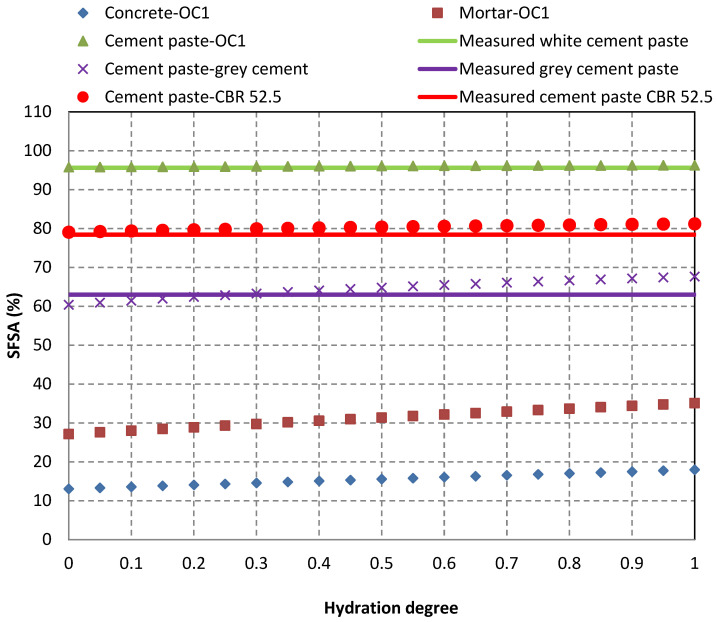
Variations of theoretical *SFSA* for pure grey cement paste, pure cement paste with CBR CEM I 52.5 N and for pure white cement-based paste, mortar of concrete OC1 and concrete OC1. Three horizontal solid lines show the comparison with the values obtained for the corresponding cement pastes.

Table 6 presents the required values to calculate the *SFSA* for the three tested cements (white cement CEM I 52.5 N, grey cement CEM II/A-L 52.5 N and CBR CEM I 52.5 N). Figure 7 demonstrates the variation of the *SFSA* for the three pure cement pastes, the mortar of concrete OC1 and concrete OC1 itself. The measured experimental *SFSA* values on cement pastes are also shown with three horizontal lines (Figure 7). The experimentally obtained *SFSA* values were close to the calculated values for the three pure cement pastes. Thus, the measurement of *SFSA* allowed an accurate estimation of the *HCPC* in the case of white cement (*SFSA* is close to 1 for pure white cement paste). However, for a grey cement paste based on cement CEM II/A-L 52.5 N and cement paste based on cement CBR CEM I 52.5 N, the *SFSA* remained below the *HCPC*.

#### 3.1.4. Hardened Cement Paste Content in RCA

Table 7 shows the obtained *SFSA* values for laboratory-produced RCA (four granular classes) and for the fraction 0/5 mm. The *SFSA* value of full fraction 0/5 mm was calculated by the obtained *SFSA* values of each granular class and the mass percentage of each fraction. The experimentally obtained *SFSA* was compared with the theoretically calculated value from the mortar and concrete composition of the initial concrete, whereas the degree of hydration *α* at 28 days and 90 days were considered as 0.7 and 0.9, respectively.

The results indicated that *SFSA* increased as the particle size decreased (Table 7), whatever the concrete composition and the degree of hydration. The *SFSA* of RCAl_OC1 for the fraction 0/0.63 mm was 26.54%, while it was 19.35% for the fraction 2.5/5 mm. Etxeberria et al. [8] stated that the finer fraction of RCA had higher adherent mortar content (it was 20% for fraction 10/25 mm and 40% for fraction 4/10 mm). De Juan and Gutiérrez [25] mentioned that the adherent mortar content was higher as the fraction was lower. Thus, the experimental results obtained in this study were in agreement with the tendency obtained from the literature [8,25]. A reasonable linear trend with the correlation coefficient R^2^ between 0.82 and 0.99 was found between the *SFSA* and average particle size for all concretes. Moreover, all experimental values were within the range of the calculated values from mortar and concrete composition whatever the granular fraction. This demonstrated that the smaller particles (mainly crushed mortar and coarse natural aggregates from parent concrete) were generated when the crushing procedure was used for the production of RCA [25].

For all studied RCA, the *SFSA* values obtained for RCA_90 were slightly higher than that of RCA produced from the same concrete at the age of 28 days. This result was confirmed by the previous theoretical calculation (the *SFSA* increased with the degree of hydration for the RCA made from mortar OC1 and concrete OC1).

In addition, the results showed that *SFSA* was highly related to the composition of initial concrete. The *SFSA* of RCA_OC1 was lower than that of RCA_OC2 obtained from concrete OC2 containing a higher cement paste volume and the same W/C ratio. Similarly, the *SFSA* of RCA_OC2 was lower than that of RCA_OC3 obtained from concrete OC3 which contained the same volume of a denser cement paste (lower W/C ratio). Thus, the *SFSA* depended closely on the quantities of cement and aggregates used in the parent concrete (as shown in Equation (14)).

**Figure 8:**
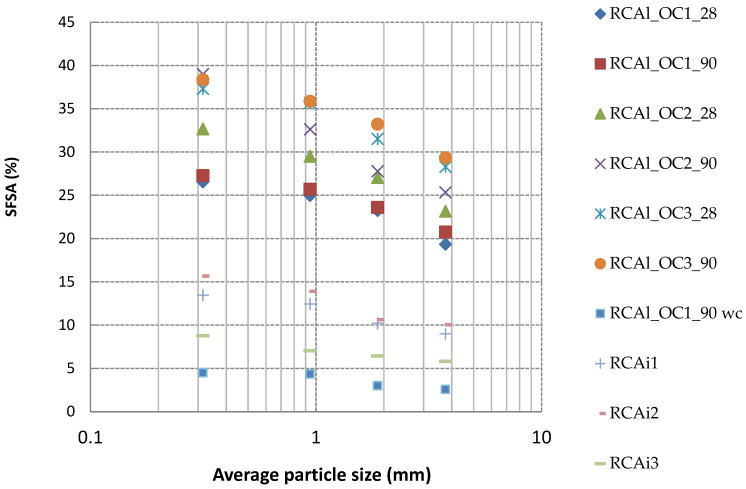
*SFSA* as a function of the average particle size of the four granular classes for all studied RCA.

Figure 8 reports the variation of *SFSA* vs. the granular classes for all studied RCA. A quasi-linear relationship between the *SFSA* and granular class (correlation coefficient R^2^ between 0.77 and 0.99) was obtained. The *SFSA* obtained for industrial RCA was significantly lower than those measured for laboratory-manufactured RCA. The *SFSA* obtained for all laboratory-manufactured RCA was in the range of 20–40%, while it was in the range of 5–15% for the industry RCA. These results might be attributed to the chemical and mineralogical composition of cement used in the manufacture of the initial concrete (probably CEM II containing a higher insoluble fraction such as limestone in salicylic acid) and any carbonation of cement paste could also reduce the soluble fraction [12]. The values obtained for industrial RCA were between the values of noncarbonated laboratory-produced RCA and carbonated laboratory-produced RCA (for example the *SFSA* of RCAl_OC1_90 was 27% for fraction 0/0.63 mm and it was 5% for RCAl_OC1_90 wc). The industrial RCA were partly carbonated, the carbonated phases cannot dissolve in salicylic acid [12] and, therefore, lower *SFSA* values were obtained for the three industrial RCA compared with noncarbonated laboratory-produced RCA.

### 3.2. Relationships between *HCPC* and the Other Properties

#### 3.2.1. Relationship between *HCPC* and Density

**Figure 9:**
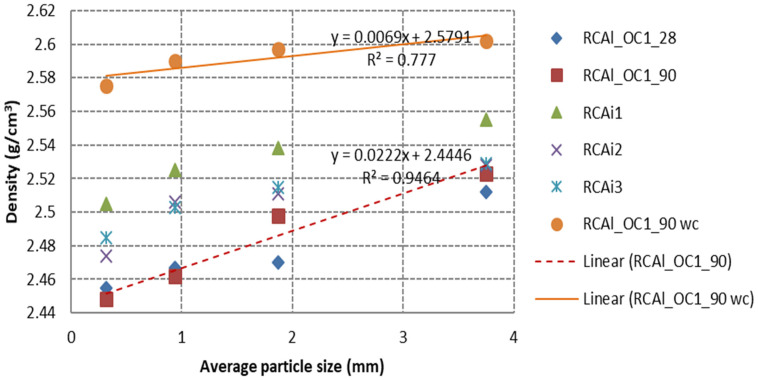
Density as a function of the granular classes for all studied RCA.

Figure 9 illustrates the variation of density as a function of granular classes. The density of RCA linearly increased as the particle size of RCA increased, which was attributed to the presence of higher *HCPC* in the finer fraction of RCA. The density of all the fractions of RCA was lower than that of natural aggregates, which was due to the lower density of hardened cement paste than that of natural aggregates [12,25]. The density of all the fractions of RCAl_OC1_90 wc was higher than that of RCAl_OC1_90. This result could be attributed to the transformation of portlandite to calcite, which presented a higher density than portlandite. After the treatment of accelerated carbonation, the slope of density to average particle size was lower than that of RCAl_OC1_90 (0.007 for RCAl_OC1_90 wc and 0.022 for RCAl_OC1_90, respectively), it can be explained that the finer fraction of RCA has higher *HCPC*, and the density of the latter was increased by the treatment of accelerated carbonation [57,58]. Since the industrial RCA was partly carbonated, the carbonation degree was much greater than the laboratory-produced noncarbonated RCA, the higher values of density were obtained for the industrial RCA, which was attributed to the partial carbonation during the service life of concrete products and the storage of RCA in recycling center; however, other unknown parameters such as the composition of RCA, the density of NA and hardened cement paste in RCA could also affect the density of industrial RCA.

**Figure 10:**
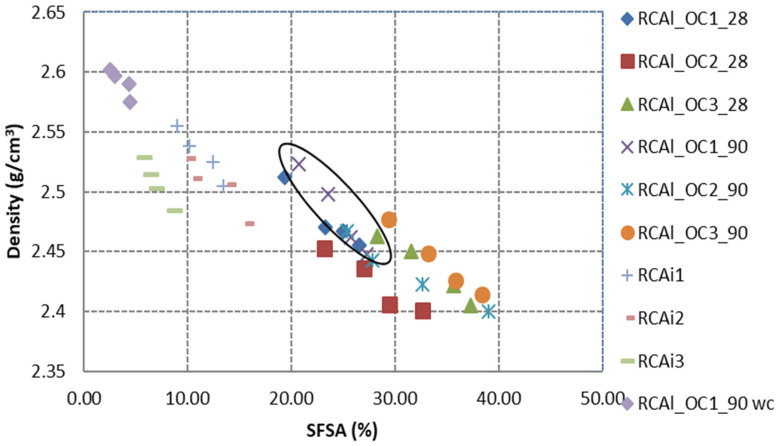
Correlation between specific density and *SFSA* in RCA.

Figure 10 presents the variation of specific density as a function of *SFSA*. The specific density decreased linearly when the *SFSA* increased. Indeed, the density of RCA directly depended on the proportion of hardened cement paste and the density of hardened cement paste and natural aggregates. If we considered the intersection points of these relations with the axis of coordinates (x = 0), we can obtain the density of the natural aggregates (NA). As shown in Table 8, the density of NA obtained for all RCA was in the range of 2.6–2.77 g/cm^3^, which signified the calculation through the relations seems to be reasonable. In the same way, when all the RCA was composed of hardened cement paste (x = 100%), we can obtain the density of hardened cement paste. As shown in Table 8, the density of hardened cement paste of RCAi3 was less than that of other materials. It has to be noted that, for the industrial RCA studied (as well as for well-carbonated RCA: RCAl_OC1_90 wc), the range of variation of *SFSA* was very limited (about 5%) which certainly led to large uncertainties on the extrapolated values of NA and hardened cement paste. The correlation coefficients between density and *SFSA* (R^2^) were within the range of 0.7 to 0.96. The slope of density to *SFSA* changed a little (from −0.012 to −0.011 after the carbonation; the black circle shows the RCA before carbonation in Figure 9).

#### 3.2.2. Relationship between *HCPC* and Water Absorption

Table 9 shows the water absorption determined with two methods (EN and IFSTTAR) for all studied RCA. For the granular fractions greater than 0.63 mm, the water absorption values obtained by these two methods were very close to each other (the absolute difference was less than 1% for all studied RCA). These results showed that the two protocols can both be used to correctly estimate the saturated surface dry (SSD) state of coarse particles greater than 0.63 mm and thus the water absorption of fractions greater than 0.63 mm.

For the finer fraction 0/0.63 mm, the EN method did not allow us to precisely identify the SSD state. The small angular grains may form some cohesion even if all the water on the surface of particles were removed, which prevented the collapse of the grains once the cone was removed [49]. Therefore, the standard EN protocol underestimated the water absorption for a fraction less than 0.63 mm.

On the contrary, the water absorption increased for the finer fraction with the IFSTTAR method (Table 9). Absorbent paper allowed for drying the surface of the fine particles, but the agglomerates were not able to be broken during the drying by absorbent paper because of capillary forces [56]. Therefore, the IFSTTAR method overestimated the water absorption of the fraction of RCA less than 0.63 mm. The same trends were obtained for laboratory-manufactured RCA and industrial RCA.

As can be seen in Table 9, the water absorption obtained with all the granular classes of well-carbonated RCA (RCAl_OC1_90 wc) was significantly lower than those obtained with noncarbonated RCA (RCAl_OC1_90). It can probably be attributed to the reduction in porosity caused by the transformation of portlandite to calcite in hardened cement paste during the accelerated carbonation [59,60,61].

**Figure 11:**
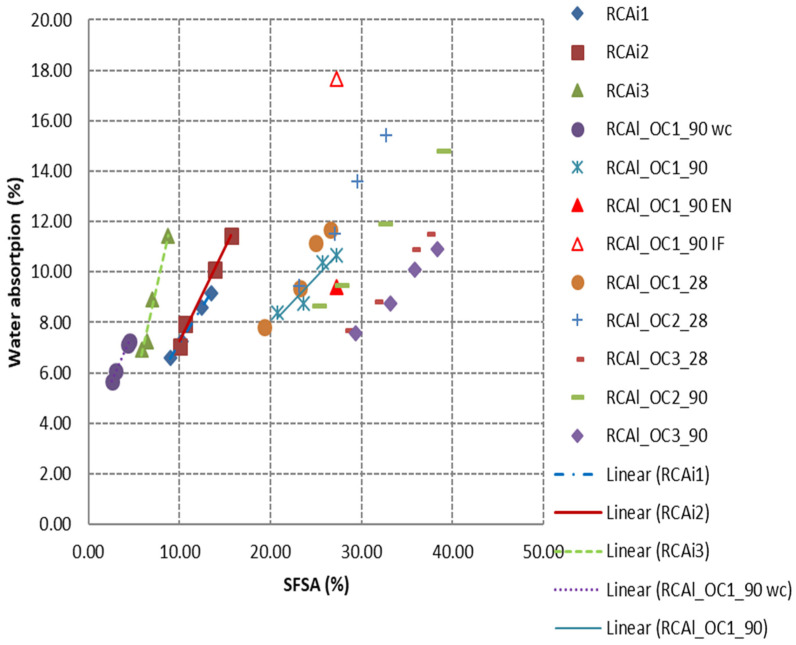
Correlation between water absorption (IFSTTAR method) and *SFSA* for all studied RCA.

Figure 11 demonstrates the water absorption (IFSTTAR method) as a function of the *SFSA*. The water absorption increased linearly with the increase in *SFSA*. The water absorption measured by the EN or IFSTTAR method for the finer fraction (0/0.63 mm) seems to be either underestimated or overestimated, respectively (for example the fraction 0/0.63 mm of RCAl_OC1_90 is shown in Figure 11). The water absorption of RCA depended on the water absorptions of hardened cement paste and NA and the proportions of hardened cement paste [49]. For a given initial concrete composition, the water absorptions of natural aggregates (*WA_NA_*) and the hardened cement paste (*WA_CP_*) did not depend on the granular classes of RCA. Thus, the water absorption of a given granular fraction of RCA (*WA_RCA_*) can be determined with Equation (15).

(15) $${WA}_{RCA}={WA}_{CP}\times HCPC+{WA}_{NA}\times (1-HCPC)$$

where *HCPC* is the hardened cement paste content in RCA for the considered granular class.

Equation (15) indicated that *WA_RCA_* varied linearly with the *HCPC*. Thus, the water absorption of the finer fraction (0/0.63 mm) can be obtained by extrapolation of the relation between *WA* and *SFSA* determined with the three coarser fractions of RCA. The extrapolation carried out using both EN and IFSTTAR methods gave similar values for the water absorption of fraction 0/0.63 mm. The average difference between these two values obtained for all studied RCA was 0.94% (Table 10). As expected, the water absorption of finer fraction obtained by extrapolation was between the value obtained by the EN and IFSTTAR methods (the water absorption of the fraction 0/0.63 mm corresponds to the extrapolated values from experimental results with the IFSTTAR method in Figure 11; the values measured by the EN and IFSTTAR methods were also reported for RCAl_OC1_90 to demonstrate that these experimental values are not appropriate). The accurate total water absorption of RCA used (fraction 0/5 mm) can be determined by knowing the proportion of each fraction through sieve analysis and its water absorption.

As shown in Figure 11, all industrial RCA tested were composed of the range of laboratory-produced RCA and well-carbonated RCA. As discussed previously, the water absorption of RCA directly depended on the water absorptions of hardened cement paste and NA, and the hardened cement paste content (*HCPC*) could also affect it. The water absorption of NA can be neglected compared with the water absorption of hardened cement paste. The water absorption of hardened cement paste depended on the property or porosity of hardened cement paste. Therefore, the water absorption of RCA depended on the properties of hardened cement pastes (W/C ratio, nature of cement, carbonation state) and *HCPC*. Thus, drawing the relationship between water absorption and the *SFSA* can be a very convenient way to differentiate different sources of RCA, since the composition of parent concrete is generally unknown for industrial RCA obtained from the recycling center. The slope of this regression can also be used to estimate the effect of weathering or some specific treatment of RCA after being crushed in a factory. Indeed, the linear relationship between the *HCPC* (obtained through *SFSA*) and the average particle size will be remained, because the presence of insoluble phases of the hardened cement paste will impact similarly for all granular classes of a given RCA. On the other hand, insoluble phases will impact the coefficients of the linear regression of the water absorption as a function of *HCPC* as this latter will decrease with an increase in the content of insoluble phases.

The method based on the dissolution of the major part of the hardened cement paste contained in RCA by salicylic acid (*SFSA*) seemed to be also applicable for the measurement of *HCPC* of industrial RCA. The method was not applied to obtain the absolute *HCPC* of industrial RCA, especially for carbonated RCA, as salicylic acid cannot dissolve the carbonated phases from hardened cement paste. However, the insoluble phases of the hardened cement paste will similarly impact all the granular classes. Therefore, it can give the slope of *SFSA* with granular class, and the relationship between water absorption and *SFSA* can be used to extrapolate the water absorption of the finer fraction for industrial RCA.

## 4. Conclusions

A method to estimate the hardened cement paste content in RCA based on the dissolution in a solution of salicylic acid in methanol was validated through a theoretical approach. It proved to be applicable for the measurement of *HCPC* for industrial RCA and laboratory-manufactured RCA. The main conclusions can be drawn as follows:

(1) The XRD results confirmed that the salicylic acid allowed us to dissolve most of the phases contained in OPC cement paste (Ca(OH)_2_, C-S-H, ettringite, *C*_2_*S*, *C*_3_*S*) but not the main phases contained in natural aggregates and especially limestone;
(2) The experimental results showed that 95.57% of white cement paste and 62.99% of grey cement paste were dissolved while only 0.83% of siliceous sand and 3.21% of calcareous aggregate were dissolved. For white cement paste, the soluble fraction in salicylic acid (*SFSA*) was almost identical to the *HCPC*. For the grey cement paste (higher content of *C*_3_*A*, *C*_4_*AF* and calcite), *SFSA* corresponded to only 62.99% of *HCPC*. For cement paste made with CBR CEM 52.5 N after a long time cured in water, *SFSA* corresponded to 78.42% of *HCPC*. Most phases of cement paste made with blend cement (CBR CEM III/A 42.5 N: Portland cement combined with slag) can dissolve in salicylic acid, which corresponded to 80.14% of *HCPC*;
(3) The difference between *SFSA* and *HCPC* could be estimated theoretically by modelling the hydration of cement paste with the chemical equations and the corresponding stoichiometric ratios. The experimentally obtained *SFSA* values were very close to the values calculated for the three pure cement pastes. The model results were consistent with the experimental results;
(4) *SFSA* in RCA obtained from industrial center increased as the particle size decreased, which showed a similar tendency as laboratory-manufactured RCA. In addition, all experimental values were within the range of the calculated values based on mortar and concrete composition. This indicated that the RCA contained both crushed mortar and part of coarse NA from parent concrete. The *SFSA* obtained for industrial RCA was significantly lower than those measured for laboratory-manufactured RCA. These results might be attributed to the nature of cement used in parent concrete (e.g., CEM II contains a higher insoluble fraction in salicylic acid such as limestone) and any carbonation of cement paste also reducing the soluble fraction. Therefore, *SFSA* did not give the exact value of *HCPC* for industrial RCA, but it was demonstrated that the *SFSA* was sufficient to correlate *HCPC* with the other properties of RCA.
(5) The properties of RCA including specific density, water absorption and porosity were strongly correlated to *HCPC*. The higher the *HCPC* or *SFSA*, the higher the water absorption and porosity, and the lower the specific density. The linear relationship can be obtained between the water absorption (porosity, specific density) and the *HCPC* or *SFSA*. The water absorption could be estimated with good accuracy for very fine RCA (laboratory-manufactured RCA or industrial RCA) by extrapolating the relationship obtained between water absorption and *HCPC* or *SFSA* with coarser granular class, which was important for the formulation of concrete made with RCA, but the value was quite difficult to accurately measure.

## Figures and Tables

**Table 1:** Summary of the hardened cement paste content or adherent mortar content measurement of RCA from literature.

Author (Reference No.)	Test Methods	Fractions of RCA (mm)	Adherent Mortar or Hardened Cement Paste Content (%)	Advantages/Disadvantages
Etxeberria et al. [8]	Not mentioned	CRCA 4/10, 10/25	40% for fraction 4/10; 20% for fraction 10/25	-
De Juan et al. [25]	Thermal method	15 samples of CRCA 4/8, 8/16	33–55% for fraction 4/8; 23–44% for fraction 8/16	This method is only suitable for CRCA because the removal of mortar necessitates “brushing” the RCA, which is difficult with small particles.
Nagataki et al. [32]	Hydrochloric acid solution method	CRCA 5/20	52.3–55% for level 1; 30.2–32.4% for level 3	This method cannot be used for RCA containing limestone aggregates and filler, which are also dissolved by hydrochloric acid.
Yagishita et al. [31]	Hydrochloric acid solution method	CRCA 5/10, 10/20	40.2% for low-grade fraction 10/20, 35.2 for low-grade fraction 5/10; 26% for medium-grade fraction 10/20, 16.7 for low-grade fraction 5/10	
Abbas et al. [26]	Image analysis method and sodium sulphate solution method	Two CRCA 4.75/9.5, 9.5/12.7,12.7/19	Image analysis: 30%, 21%, 21% for 4.75/9.5, 9.5/12.7, 12.7/19, respectively; Sodium sulphate solution method: 26%,22%,21% for 4.75/9.5, 9.5/12.7, 12.7/19, respectively	The image analysis method is suitable for the quantification of residual mortar in CRCA. Moreover, this method is long to perform as a statistical approach is needed. The sodium sulphate solution method is only suitable for CRCA.
Hansen and Narud [28]	Linear traverse method	CRCA 4/8, 8/16, 16/32	58–64% for fraction 4/8; 38–39% for fraction 8/16; 25–35% for fraction 16/32	This linear traverse method is only suitable for adherent mortar content of CRCA.
Topçu et al. [27]	Linear traverse method	CRCA 4/8, 16/32	60% for fraction 4/8; 30% for fraction 16/32	
Ulsen et al. [29]	X-ray SEM-based image analysis	FRCA 0.15/3.0	*HCPC*: 10% for the fraction 0.15/3.0; 15% for the fraction 0.15/0.3	The correlations between the sum of CaO and LOI and cement paste + carbonates, and the comparison to the cement plus carbonate content by HCl leaching stated the reliability SEM-based image analysis.
Macedo et al. [30]	The dissolution method in deionized water (10 days)	Simulated 1%, 5%, 10% and 20% hardened cement paste	The deionized method presents good efficiency in the removal of the calcium ions from the hydrated cement phases, C-S-H and CH in the simulated samples	There is the carbonation contamination and dissolution in deionized water takes ten days, which is too long. In addition, the ICP analysis is expensive.

**Table 2:** Mineralogical composition of cement determined by the XRD-Rietveld (%).

	*C*_3_ *S*	*C*_2_ *S*	*C*_3_ *A*	*C*_4_ *AF*	Anhydrite	Calcite	Periclase	Gypsum	Quartz	Slag
White cement CEM I 52.5 N	73.90	21.87	1.46	-	0.52	1.53	0.72	-	-	-
Grey cement CEM II/A-L 52.5 N	52.37	8.01	8.86	8.89	0.74	17.93	0.46	2.05	0.7	-
CBR CEM III/A 42.5 N	35.1	7.91	3.29	5.22	0.16	0.03	-	0.86	-	44.01
CBR CEM I 52.5 N	66.97	12.08	7.19	9.47	0.02	1.03	-	1.76	-	-

**Table 3:** XRD results before and after dissolution in salicylic acid and methanol for cement and pastes (number of * refers to the percentage of phases, the more *, the more percentage presented).

Sample	Insoluble Phases
1. After the dissolution of white cement (Figure 2)	Calcite Ca(CO_3_) ****,
	Calcium Sulfate Ca(SO_4_) **,
	Syngenite K_2_Ca(SO_4_)_2_.H_2_O *,
	Calcium Sulfate Hydrate Ca(SO_4_)(H_2_O)_0.5_ *,
	Calcium Aluminum Oxide Ca_3_Al_2_O_6_ ***,
	Calcite, magnesian Ca,Mg(CO_3_) **
2. After the dissolution of grey cement (Figure 3)	Calcite Ca(CO_3_) ****,
	Brownmillerite Ca_2_(Al, Fe ^+ 3^)_2_O_5_ **,
	Anhydrite Ca(SO_4_) **,
	Gypsum Ca(SO_4_).2H_2_O *, Quartz SiO_2_ *,
	Calcium Aluminum Oxide Ca_3_Al_2_O_6_ ***
	Calcite, magnesian Ca,Mg(CO_3_) *
3. Before the dissolution of white cement paste (Figure 4)	Portlandite Ca(OH)_2_ ****, β-dicalcium Silicate Ca_2_SiO_4_ **,
	Calcite Ca(CO_3_) *, Tricalcium Silicate Ca_3_SiO_5_ *,
	Calcium Silicate Hydrate Ca_1.5_SiO_3.5_ ×H_2_O *
4. After the dissolution of white cement paste (Figure 4)	Calcite Ca(CO_3_) ****,
	Calcium Sulfate Hydrate Ca(SO_4_).0.5H_2_O ***,
	Bassanite Ca(SO_4_).0.5H_2_O ***
5. Before the dissolution of grey cement paste (Figure 5)	Portlandite Ca(OH)_2_ ****, Calcite Ca(CO_3_) ***, Quartz SiO_2_ *,
	β-dicalcium Silicate Ca_2_SiO_4_ **,
	Calcium Aluminum Oxide Carbonate Hydroxide Hydrate (AFm hemi carbonate) Ca_4_Al_2_O_6_(CO_3_)_0.5_(OH).11.5H_2_O **,
	Brownmillerite Ca_2_(Al, Fe ^+3^)_2_O_5_ *
6. After the dissolution of grey cement paste(Figure 5)	Calcite Ca(CO_3_) ****, Quartz SiO_2_ *
	Calcium Aluminum Oxide Ca_3_Al_2_O_6_ *,
	Brownmillerite Ca_2_(Al, Fe ^+ 3^)_2_O_5_ **,
	Calcium Sulfate Hydrate Ca(SO_4_).0.5H_2_O *,
	Calcium Aluminum Iron Oxide Ca_3_(Al, Fe)_2_O_6_ *
7. Before the dissolution of RCAl_OC1_90 0.63/1.25 mm (Figure 6)	Calcite Ca(CO_3_) ****, Quartz SiO_2_ **,
	Portlandite Ca(OH)_2_ **
8. After the dissolution of RCAl_OC1_90 0.63/1.25 mm (Figure 6)	Calcite Ca(CO_3_) ****, Quartz SiO_2_ **,
	Dolomite CaMg(CO_3_)_2_ *

**Table 4:** Experimental results of *SFSA* for cement pastes and natural aggregates (%).

	Test 1	Test 2	Test 3	Mean Value	Standard Deviation Value
White cement paste (CEM I 52.5 N)	95.46	96.35	94.89	95.57	0.74
Grey cement paste (CEM II/A-L 52.5 N)	62.56	63.08	63.33	62.99	0.39
Cement paste (CBR CEM III/A 42.5 N)	79.87	80.09	80.48	80.14	0.31
Cement paste (CBR CEM I 52.5 N)	78.29	78.26	78.70	78.42	0.24
Siliceous sand	0.76	0.86	0.88	0.83	0.06
Calcareous aggregate	3.42	3.03	3.18	3.21	0.20

**Table 5:** Chemical equations and corresponding stoichiometric ratios used for the modelling of hydration of cement.

Chemical Equations	Stoichiometric Ratios
$C_{3}S+5.5H\to C_{1.7}SH_{4.2}+1.3CH$	$\left(E/C_{3}S\right)=0.434$
$C_{2}S+4.5H\to C_{1.7}SH_{4.2}+0.3CH$	$\left(E/C_{2}S\right)=0.471$
$C_{3}A+3C\bar{S}H_{2}+26H\to C_{6}A{\bar{S}}_{3}H_{32}\left(Ett\right)$	${\left(\left(E+C\bar{S}H_{2}\right)/C_{3}A\right)}_{Ett}=3.644$
$C_{3}A+C\bar{S}H_{2}+10H\to C_{4}A\bar{S}H_{12}\left(AFm\right)$	${\left(\left(E+C\bar{S}H_{2}\right)/C_{3}A\right)}_{AFm}=1.304$
$C_{4}AF+3C\bar{S}H_{2}+30H\to CH+FH_{3}+C_{6}A{\bar{S}}_{3}H_{32}\left(Ett\right)$	${\left(\left(E+C\bar{S}H_{2}\right)/C_{4}AF\right)}_{Ett}=2.173$
$C_{4}AF+C\bar{S}H_{2}+14H\to CH+FH_{3}+C_{4}A\bar{S}H_{12}\left(AFm\right)$	${\left(\left(E+C\bar{S}H_{2}\right)/C_{4}AF\right)}_{AFm}=0.872$
$C\bar{S}+2H\to C\bar{S}H_{2}$	$\left(E/C\bar{S}\right)=0.265$

**Table 6:** Values needed to calculate the *SFSA* for the three cements.

	*x*	*y*	*Csol*	*(E*/*C)sol*	*(E*/*C)moy*
White cement (CEM I 52.5 N)	0.9775	0.9797	0.9614	0.4370	0.4527
Grey cement (CEM II/A-L 52.5 N)	0.8092	0.7462	0.6482	0.3940	0.5408
CBR CEM I 52.5 N	0.9749	0.8109	0.8322	0.4645	0.5968

**Table 7:** *SFSA* for different granular classes of the laboratory-produced RCA (unit: %, the numbers 28 and 90 refer to the aging of the original concrete samples in days).

Fractions (mm)	RCAl_OC1_28	RCAl_OC1_90	RCAl_OC2_28	RCAl_OC2_90	RCAl_OC3_28	RCAl_OC3_90
0/0.63	26.54	27.28	32.66	39.01	37.31	38.36
0.63/1.25	24.98	25.72	29.51	32.63	35.68	35.86
1.25/2.5	23.25	23.60	27.06	27.79	31.53	33.22
2.5/5	19.35	20.76	23.16	25.35	28.29	29.34
0/5	22.58	23.35	26.55	29.27	31.59	32.63
Calculated value on concrete	16.53	17.48	21.36	22.50	25.90	27.20
Calculated value on mortar	32.90	34.38	40.10	41.70	46.16	47.80

**Table 8:** Coefficients of the linear relationships between density and *SFSA* (y = ax + b).

	a	b	R^2^	Density of NA (x = 0)	Density of Hardened Cement Paste (x = 100%)
RCAi1	−0.010	2.64	0.95	2.64	1.64
RCAi2	−0.008	2.60	0.82	2.60	1.84
RCAi3	−0.014	2.61	0.96	2.61	1.17
RCAl_OC1_90	−0.011	2.63	0.78	2.63	1.55
RCAl_OC1_90 wc	−0.012	2.77	0.98	2.77	1.57

**Table 9:** Water absorption of all fractions (in mm) of studied RCA determined by standard EN (*WA*_EN_) and IFSTTAR (*WA*_IF_) methods (%).

	*WA*_EN_				*WA*_IF_			
	0/0.63	0.63/1.25	1.25/2.5	2.5/5	0/0.63	0.63/1.25	1.25/2.5	2.5/5
RCAl_OC1_28	7.61	10.46	8.22	7.76	21.90	11.15	9.36	7.83
RCAl_OC2_28	8.05	12.78	10.90	9.33	23.18	13.59	11.52	9.45
RCAl_OC3_28	9.74	10.07	8.10	7.68	21.44	10.89	8.84	7.71
RCAl_OC1_90	9.42	9.37	7.79	7.12	17.66	10.36	8.76	8.39
RCAl_OC2_90	9.77	11.02	8.67	7.99	22.84	11.94	9.50	8.66
RCAl_OC3_90	6.52	8.75	7.31	6.67	16.79	10.08	8.76	7.58
RCAl_OC1_90 wc	6.30	6.27	5.57	5.25	13.10	7.14	6.05	5.64
RCAi1	6.14	8.01	7.03	6.43	16.76	8.59	7.23	6.61
RCAi2	8.62	9.17	7.65	6.70	21.33	10.10	7.92	7.05
RCAi3	6.32	8.09	6.99	6.66	15.85	8.91	7.24	6.92

**Table 10:** Extrapolated water absorption of Fraction 0/0.63mm from standard EN and IFSTTAR for industrial RCA and laboratory-manufactured RCA.

	Tested Value of IFSTTAR (%)	Tested Value of EN 1097-6 (%)	Extrapolated Value of IFSTTAR (%)	Extrapolated Value of EN 1097-6 (%)	Difference between Two Extrapolated Values (%)
RCAi1	16.76	6.14	9.16	8.48	0.68
RCAi2	21.33	8.62	11.44	10.23	1.21
RCAi3	15.85	6.32	11.44	9.94	1.05
RCAl_OC1_90	17.66	9.42	10.67	9.82	0.85
RCAl_OC1_90 wc	13.1	-	7.26	6.35	0.91

## Data Availability

Not applicable.
